# The Development of a Murine Model for *Forcipomyia taiwana* (Biting Midge) Allergy

**DOI:** 10.1371/journal.pone.0091871

**Published:** 2014-03-20

**Authors:** Mey-Fann Lee, Kai-Jei Yang, Nancy M. Wang, Yung-Tsung Chiu, Pei-Chih Chen, Yi-Hsing Chen

**Affiliations:** 1 Department of Medical Research Taichung Veterans General Hospital, Taichung, Taiwan; 2 Department of Life Science, Tunghai University, Taichung, Taiwan; 3 Institute of Biotechnology, National Changhua University of Education, Changhua, Taiwan; 4 Division of Allergy, Immunology and Rheumatology, Taichung Veterans General Hospital, Taiwan; 5 Faculty of Medicine, National Yang-Ming University, Taipei, Taiwan; Institute for Virus Research, Laboratory of Infection and Prevention, Japan

## Abstract

**Background:**

*Forcipomyia taiwana* (biting midge) allergy is the most prevalent biting insect allergy in Taiwan. An animal model corresponding to the human immuno-pathologic features of midge allergy is needed for investigating the mechanisms and therapies. This study successfully developed a murine model of *Forcipomyia taiwana* allergy.

**Methods:**

BALB/c mice were sensitized intra-peritoneally with midge extract on days 0, 7, 14, 21 then intra-dermally on days 28, 31 and 35. Serum midge-specific IgE, IgG1, and IgG2a were measured every 14 days by indirect ELISA. The mice were challenged intradermally with midge extract at day 40 and then sacrificed. Proliferation and cytokine production of splenocytes after stimulation with midge extract were determined by MTT assay and ELISA, respectively. The cytokine mRNA expression in response to midge stimulation was analyzed by RT-PCR.

**Results:**

Serum IgE, total IgG, and IgG1 antibody levels against midge extract were significantly higher in the midge-sensitized mice than in the control mice. After the two-step sensitization, all mice in the midge-sensitized group displayed immediate itch and plasma extravasation reactions in response to challenge with midge extract. Skin histology from midge-sensitized mice showed marked eosinophil and lymphocyte infiltrations similar to that observed in humans. Stimulation of murine splenocytes with midge extract elicited significant proliferation, IL-4, IL-10, IL-13 and IFN-γ protein production, and up-regulation of mRNA in a dose-dependent manner in the midge-sensitized group, but not in the control group.

**Conclusions:**

A murine model of midge bite allergy has been successfully developed using a two-step sensitization protocol. The sensitized mice have very similar clinical and immunologic reactions to challenge with midge proteins as the reactions of human to midge bites. This murine model may be a useful platform for future research and the development of treatment strategies for insect bite allergy.

## Introduction

*Forcipomyia taiwana* (biting midge) allergy is the most prevalent biting insect allergy in Taiwan. Nearly 60% of exposed subjects develop reactions to midge bites [1]. There are two types of reactions: 1) immediate, with large local swelling at biting sites within one hour of the bite and 2) delayed, with intense itching papules and vesicles/bullae at biting sites 6–24 hours after the bites. Delayed-type lesions may turn centrally necrotic several days later and may last for weeks or even months. Among midge-allergic individuals, 14% develop a solely immediate reaction, 43% develop an immediate reaction followed by a delayed reaction, and 43% develop solely delayed reaction [1]. Unlike mosquitoes, the blood-sucking midges are very species-specific. The female midge sucks only human blood, but not blood of other animals, for spawning purposes [2]–[4]. From our clinical observation, the reactions to midge bites are typically stronger than that of mosquito bites in the same individuals. However, some midge-allergic individuals who live in midge-prevalent areas may develop tolerance to the bites after frequent repeated bites (Chen, unpublished data).

From previous studies, immediate reactions to midge bites are IgE-mediated, while patients with delayed type reactions have lympho-histiocytes and eosinophil infiltration at biting sites and their peripheral mononuclear cells proliferate and secret significantly more interferon-gamma (IFN-γ), interleukin-6 (IL-6), and tumour necrosis factor-alpha (TNF-α) in response to midge extracts than the midge non-allergic subjects [5]. However, the mechanisms involved in the development of the midge allergy or induction of tolerance to midge bites are not fully understood. An animal model corresponding to human allergic reactions to midge bites will provide more detailed insights into the mechanisms and development of treatment strategies.

The aim of this study was to develop a murine model of midge bite allergy. To date, this is the first report of developing both immediate and delayed biting midge reactions in a murine model.

## Materials and Methods

### Human Skin Biopsy Specimens and Immuno-histochemistry (IHC)

The study was approved by the Institutional Review Board of Taichung Veterans General Hospital (IRB TCVGH NO: 921218/271). Punch skin biopsies were performed from the biting lesions of patients with delayed-type midge allergy after signing written informed consent. Biopsies were performed within 24–48 hours after natural exposure to the midge bites. Skin specimens were fixed in 10% neutral-buffered formalin overnight and processed through a routine cycle to paraffin wax embedding. The 4-μm sections were stained with haematoxylin and eosin (H and E), anti-CD4 (1∶10 dilution) (Bio SB, CA, USA), and anti-CD8 (1∶10 dilution) monoclonal antibodies (Dako, Denmark). After primary and secondary incubations, sections were then incubated with Tris-HCl (50 mM, pH7.6) containing 0.05% of 3,3′-diaminobenzidine (DAB, Sigma) and 0.02% of hydrogen peroxide (30% H_2_O_2_, Sigma).

### Collection of *F. taiwana*

Midges were collected by inhaled UV mosquito control system (FUKADAC, cat. FMT-111, China) using a special collecting device designed by our lab.

### Preparation of Whole Body *F. taiwana* Extract

One thousand midges were ground and dissolved in 5 ml phosphate buffered saline (PBS), ultrasonicated for 30 min at 4°C, and centrifuged at 8,000 g for 15 min. The supernatant was collected, filtered through a 0.22 μm filter, aliquoted, and stored at −70°C until use.

### Animals

Female 6-week-old BALB/c mice were purchased from Taiwan National Laboratory Animal Centre and raised under pathogen-free conditions. All animal experiments were reviewed and approved by the Institutional Animal Care and Use Committee of Taichung Veterans General Hospital.

### Sensitization

**Figure 1:**
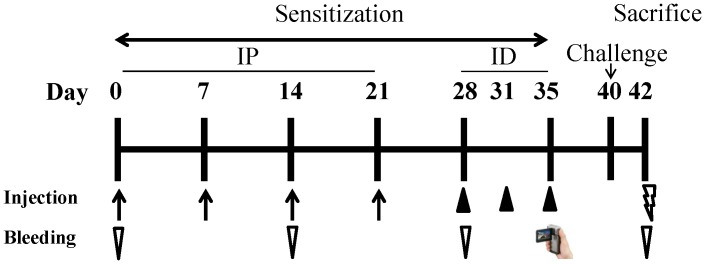
Experimental protocols. BALB/c mice were sensitized intra-peritoneally (IP) with 20 μg of midge extract (group I: midge-sensitized) or PBS (group II: controlled) on days 0, 7, 14 and 21 (n = 10 in each group). From day 28 to 35 the mice were intra-dermally (ID) sensitized with 10 μg of midge extract (group I) or PBS only (group II) on days 28, 31 and 35 (arrows). Serum samples were collected on days 0, 14, 28 and 42 as designated with inverted arrow heads. The scratching behaviours were videotaped on day 35 after the final sensitization. The mice were sacrificed on day 42.

The mice were injected intra-peritoneally (IP) with four doses of 20 μg/200 μl midge extract absorbed to 2 mg alum adjuvant on days 0, 7, 14, and 21. The mice were subsequently intra-dermally (ID) sensitized with 10 μg of midge extract in PBS on days 28, 31 and 35. The control mice were injected with phosphate buffered saline (PBS) containing alum for IP sensitization and PBS only for ID sensitization at the same time frame. Serum samples were collected from the retro-orbital venous plexus on days 0, 14, 28, 42 and stored at −20°C until analysis. The sensitization protocol was shown in Figure 1.

### Measurement of Specific Antibodies by ELISA

Specific IgE, total IgG, IgG1 and IgG2a antibodies were determined by in-house enzyme-linked immuno-sorbent assay (ELISA) with the required antibodies purchased from BD Pharmingen (San Jose, CA, USA.). Micro-titre plates were coated with midge extract for 2 hours at 37°C. After washing with PBST, the plates were blocked with 5% skimmed milk (for IgE) or 2% bovine serum albumin (BSA) (for all IgGs) for 2 h at room temperature. Sera were diluted (1∶10 for IgE or 1∶100 for all IgGs) in PBST and incubated at 4°C overnight (for IgE) or room temperature for 2 h (for all IgGs).

For IgE measurement, the plates were incubated with biotin-conjugated rat anti-mouse IgE (1∶4000) for 2 h at room temperature. Subsequently, the plates were reacted with horseradish peroxidase-conjugated streptavidin (1∶10,000) for 1 h, developed by adding TMB (Sigma), and stopped with 1 M H_3_PO_4_. For IgG measurement, the plates were incubated with horseradish peroxidase-conjugated rabbit anti-mouse IgG (1∶10,000), IgG1 (1∶5,000) or IgG2a (1∶1000) for 2 h at room temperature and developed by adding ABTS solution (Sigma). The optical density was then analyzed on a Sunrise Absorbance Reader (TECAN, Austria) at 450 nm and 415 nm, respectively.

### Immediate Allergy by Evans Blue Method

On day 40, five days after the last skin sensitization, the mice were intra-dermally challenged. A total of 150 μl 1% Evans blue was injected intravenously into the tail vein of the mice one hour before challenge. Subsequently, 20 μl of midge extracts (0.4 mg/ml) were injected intra-dermally into the shaved abdominal skin, while PBS was injected into the counterpart of the abdominal skin as a negative control. After 20 min, the mice were sacrificed and skinned. The dye was extracted from the collected skins by digestion with 150 μl 1 M KOH overnight at 37°C. The next day, 150 μl 5% H_3_PO_4_ in acetone was added and the samples were centrifuged. The supernatants were collected and measured on an Absorbance Reader (TECAN) at 620 nm to quantify the extracted dye.

### Evaluation of Scratching Behaviour

The scratching behaviours were videotaped for 1 h starting immediately after the intra-dermal challenge with midge extract. Counts of scratching were made using video playback. The observation of scratching behaviour were performed as described previously [6].

### Histologic Examination of Immediate and Delayed Type Reactions

For histologic examination, mice were intra-dermally challenged five days after the last sensitization. After 20 min, 24 h, and 48 h, the mice were sacrificed at the indicated time and the abdominal skins from the challenge sites were removed and placed in 10% formalin overnight at room temperature. The removed skin samples were 2 mm larger than the lesions, and sized varied from 4–7 mm in diameters (16–49 mm^2^). Briefly, the tissues were embedded in paraffin, cut into 5-μm sections, de-paraffinized, dehydrated, and stained with H and E. Moreover, sections were further stained with rabbit anti-CD4 (1∶800 dilution) or anti-CD8 (1∶400 dilution) polyclonal antibodies (Bioss, MA, USA). To detect CD marker positive cells, the sections were incubated with peroxidase-conjugated goat anti-rabbit IgG and stained in a substrate solution containing DAB in Bond automatic system (Leica, Newcastle, UK). Inflammatory cell infiltrates were examined by light microscopy and corresponding images were shot by the Olympus BX51 microscopic/DP71 Digital Camera System (Nagano, Japan).

### Proliferation of Splenocytes by MTT Assay

After the mice were sacrificed, their spleens were taken aseptically, minced, and filtered through sterile steel filters. The erythrocytes were lysed and the splenocytes were washed and suspended in complete RPMI medium supplemented with 10% fetal bovine sera (FBS). The cell suspensions were cultured in triplicate into 96-well round-bottomed plates at a concentration of 2×10^5^ cells/well and stimulated with 5 μg/ml Concanavalin A (ConA) or 2-fold serial dilutions of midge extract at 37°C for 44 or 68 h. Thereafter, the cultures were pulsed with 0.5 mg/ml MTT ([3-(4,5-dimethylthiazol-2-yl)-2,5-diphenyl tetrazolium bromide]) for another 4 h. After centrifugation, 100 μl DMSO were added on the cell pellets and agitated at room temperature for 15 min to dissolve the formazan dye. The cells were harvested and assayed by ELISA reader (TECAN) at 570 nm. The stimulation indices were calculated from the optical density (OD) of the specifically stimulated cells compared to the OD of non-stimulated cells.

### Measurement of Cytokine Production

Splenocytes were cultured in 24-well flat-bottomed plates at a concentration of 1×10^6^/ml and stimulated with 5 μg/ml ConA or various doses of midge extracts at 37°C for 2–5 days. The culture supernatants were collected at each time interval and stored at −20°C until the cytokine assay. Levels of IL-4, IL-6, IL-13, IL-10, and IFN-γ in the culture supernatants were measured with murine ELISA development kits (PeproTech, Rocky Hill, NJ, USA) according to the manufacturer’s instructions.

### Gene Expression by Reverse Transcription-polymerase Chain Reaction (RT-PCR)

To determine how various molecules were expressed in midge-stimulated splenocytes from treated mice, RT-PCR was done to measure cytokine expression. Pre-designed primer sequences were listed in Table 1. Briefly, cDNA were prepared from 1 μg total RNA using a SuperScript III kit (Invitrogen, Carlsbad, CA). A total volume of 50 μl of PCR mixture, which included 2 mM of MgCl_2_, 200 μM of dNTP, 1.25 U of hot-start SuperTherm Gold DNA polymerase (Hoffman-La-Roche), 10 pmole of specific sense and anti-sense primers for each cytokine gene, and 10 ng of first-strand cDNA. After beginning by a single 95°C for 10 min pre-incubation step, PCR were performed under the following conditions: denaturation, 94°C for 1 min, annealing, 60°C for 1 min, and extension for 1 min at 72°C in a thermal cycler (Perkin Elmer). The number of cycles used depended on the transcript amplified. β-actin cDNA was used as an internal control. The PCR products were analyzed with 2% agarose gel electrophoresis and ethidium bromide staining and captured by Kodak molecular imaging system (NY, USA).

**Table 1:** The sequences of gene-specific primers used in RT-PCR.

Gene (mouse)		Sequences	Produce size (bp)
**TNF-α**	F	5′ CAT CTT CTC AAA ATT CGA GTG ACA A 3′	175
	R	5′ TGG GAG TAG ACA AGG TAC AAC CC 3′	
**IL-6**	F	5′ CCA TCC AGT TGC CTT CTT G 3′	223
	R	5′ AAG TGC ATC ATC GTT GTT CAT AC 3′	
**IL-4**	F	5′ AGC CAT ATC CAC GGA TGC GAC AAA 3′	176
	R	5′ AAT ATG CGA AGC ACC TTG GAA GCC 3′	
**IL-13**	F	5′ AGA CCA GAC TCC CCT GTG CA 3′	123
	R	5′ TGG GTC CTG TAG ATG GCA TTG 3′	
**IL-10**	F	5′ CCA AGC CTT ATC GGA AAT GA 3′	155
	R	5′ AGG GGA GAA ATC GAT GAC AG 3′	
**IFN-γ**	F	5′ GGC CAT CAG CAA CAA CAT AAG CGT 3′	118
	R	5′ TGG GTT GTT GAC CTC AAA CTT GGC 3′	
**IL-5**	F	5′AGCACAGTGGTGAAAGAGACCTT 3′	117
	R	5′ TCCAATGCATAGCTGGTGATTT 3′	
**Il-6**	F	5′ CCATCCAGTTGCCTTCTTG 3′	223
	R	5′ AAGTGCATCATCGTTGTTCATAC 3′	
**IL-1β**	F	5′CTTCATCTTTGAAGAAGAGCCC 3′	418
	R	5′CTCTGCAGACTCAAACTCCAC 3′	
**β-actin**	F	5′ GGCCAACCGTGAAAAGATGA 3′	251
	R	5′ CACGCTCGGTCAGGATCTTC 3′	

### Response to Topical Glucocorticoid Treatment

In order to investigate whether the skin reactions in midge-sensitized mice could be reversed by topical glucocorticoid as observed in the clinics, topical glucocorticoid was applied on the mice on days 43–49. Five mg of carbomer base alone or 2.5 ng dexamethasone phosphate in 5 mg carbomer were applied onto the shaved abdominal skin of midge-sensitized and control mice an hour daily for 1 week. On day 50, the treated mice were intra-dermally challenged at the treated site and sacrificed after 48 hours.

### Statistical Analysis

Statistical analysis was performed with the SPSS version 12.0 software (SPSS Inc., Chicago, IL, USA) using appropriate methods. Statistical significance was set at *p*<0.05.

## Results

### H and E and Immuno-histochemical Findings in Delayed-type midge allergy

**Figure 2:**
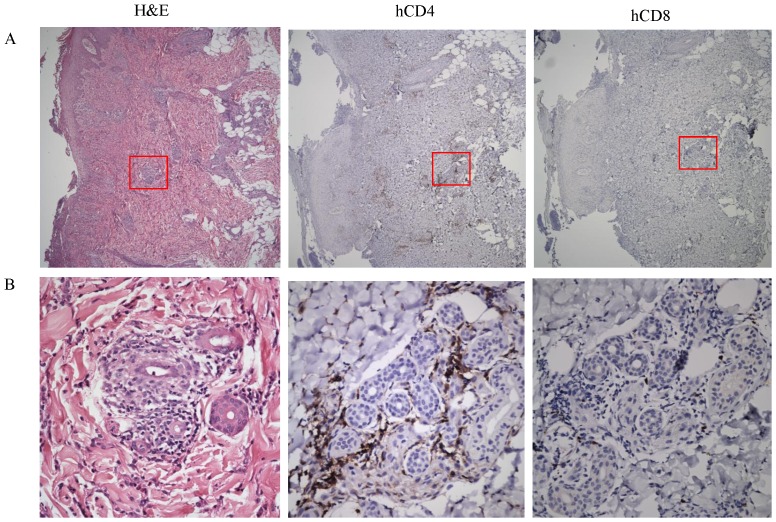
Histologic features of skin lesions in a patient with delayed-type midge allergy. Skin biopsy was performed within 48 hours after midge bites and the sections were stained with hematoxylin and eosin stain (H&E) or immuno-stained with anti-human CD4 (hCD4) or CD8 (hCD8) monoclonal antibodies. Magnification: A, ×50; B, ×400.

Skin lesions biopsied 24–48 h after midge bites from patients with delayed-type midge allergy showed spongiosis with peri-vascular lympho-histiocytes and eosinophil infiltrates spanning the upper and lower dermis (Fig. 2, panel H and E). To examine the respective contribution of CD4^+^ and CD8^+^ T cells in midge-allergic patho-physiology, immuno-cytochemical staining was performed in the same skin sections. High powered imaging showed that CD4^+^ T cells were localized predominantly in dermis of the lesion, but only very little CD8^+^ T cells were found (Fig. 2, panels hCD4 and hCD8).

### Change of Scratch Behaviour to Midge Challenge

**Figure 3:**
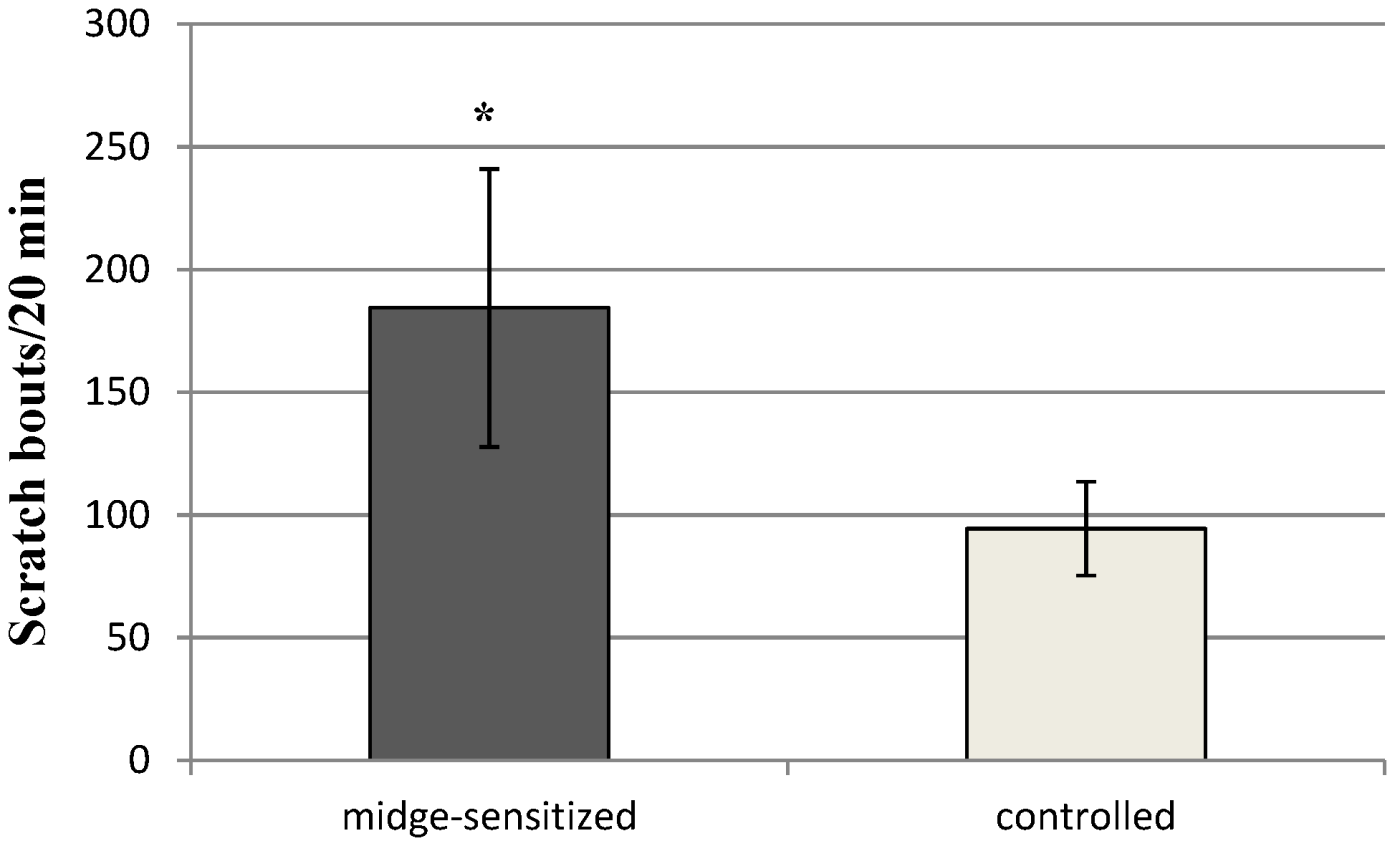
Counts of scratching bouts from midge-sensitized or controlled mice induced by intra-dermal injection of midge extract. The scratching behaviour was videotaped for 1±SEM of nine mice from each group. *p<0.05, by Mann-Whitney *U* test.

To show an immediate-allergic reaction, whether an intra-dermal injection of midge extract on the challenged region of the abdominal skin would elicit excessive scratching in sensitized mice was examined. Midge-sensitized mice demonstrated significantly more scratch bouts on the challenge sites than the control mice (*p*<0.05) (Fig. 3).

### Midge-specific Antibodies

**Figure 4:**
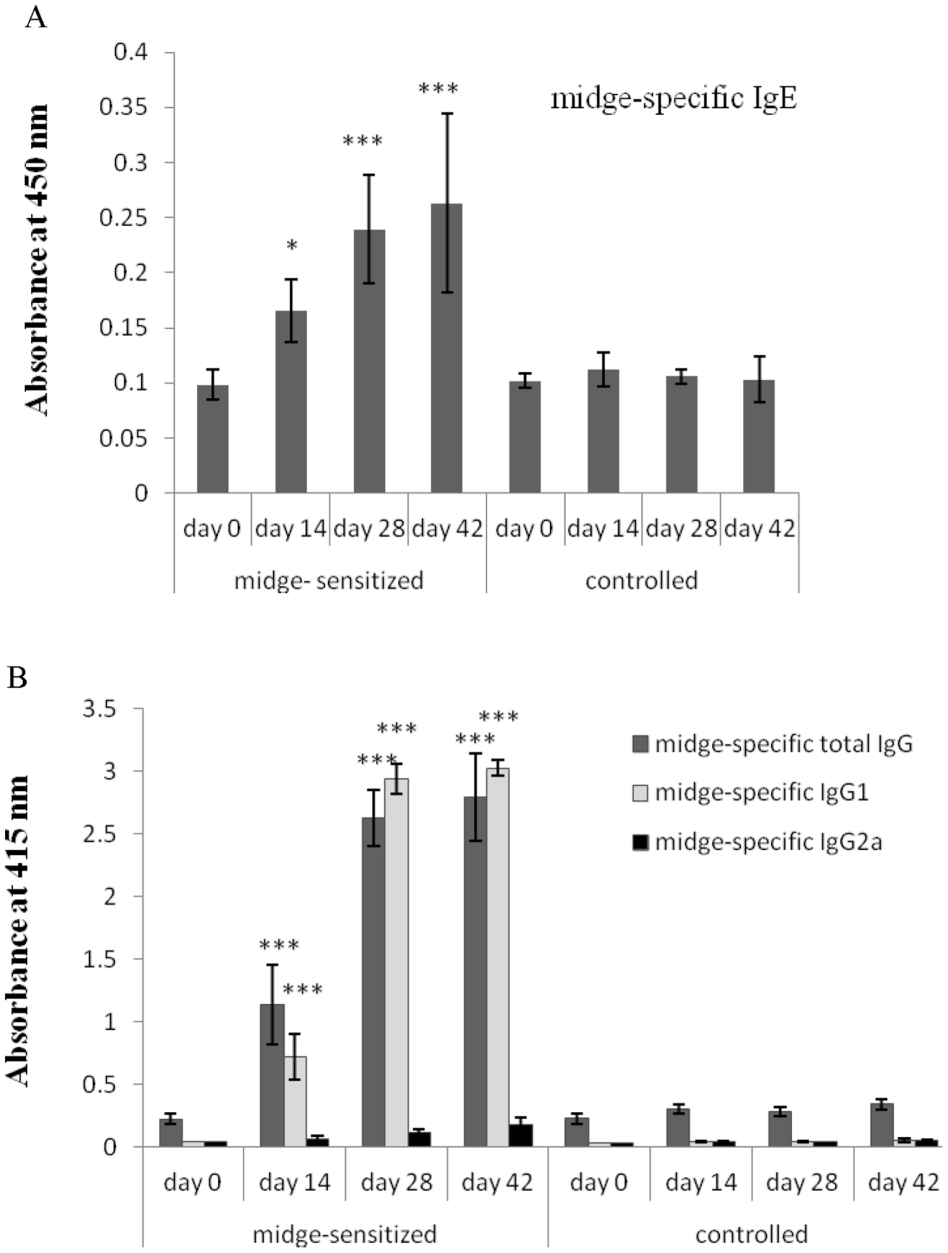
Specific antibody levels in midge-sensitized or controlled mice. (A) Midge-specific IgE and (B) total IgG, IgG1, and IgG2a antibodies in the sera at the indicated days. Results are means OD±SD (n = 10 per group). *p<0.05; ***p<0.001, by one-way ANOVA with the Dunnett *t* test.

Serum IgE, total IgG, and IgG1 antibody levels against midge, but not midge-specific IgG2a, were significantly higher in the midge-sensitized group compared to the controls since the second week of sensitization in a time-dependent, manner (Fig. 4).

### Immediate Reactions after Skin Challenge Test

**Figure 5:**
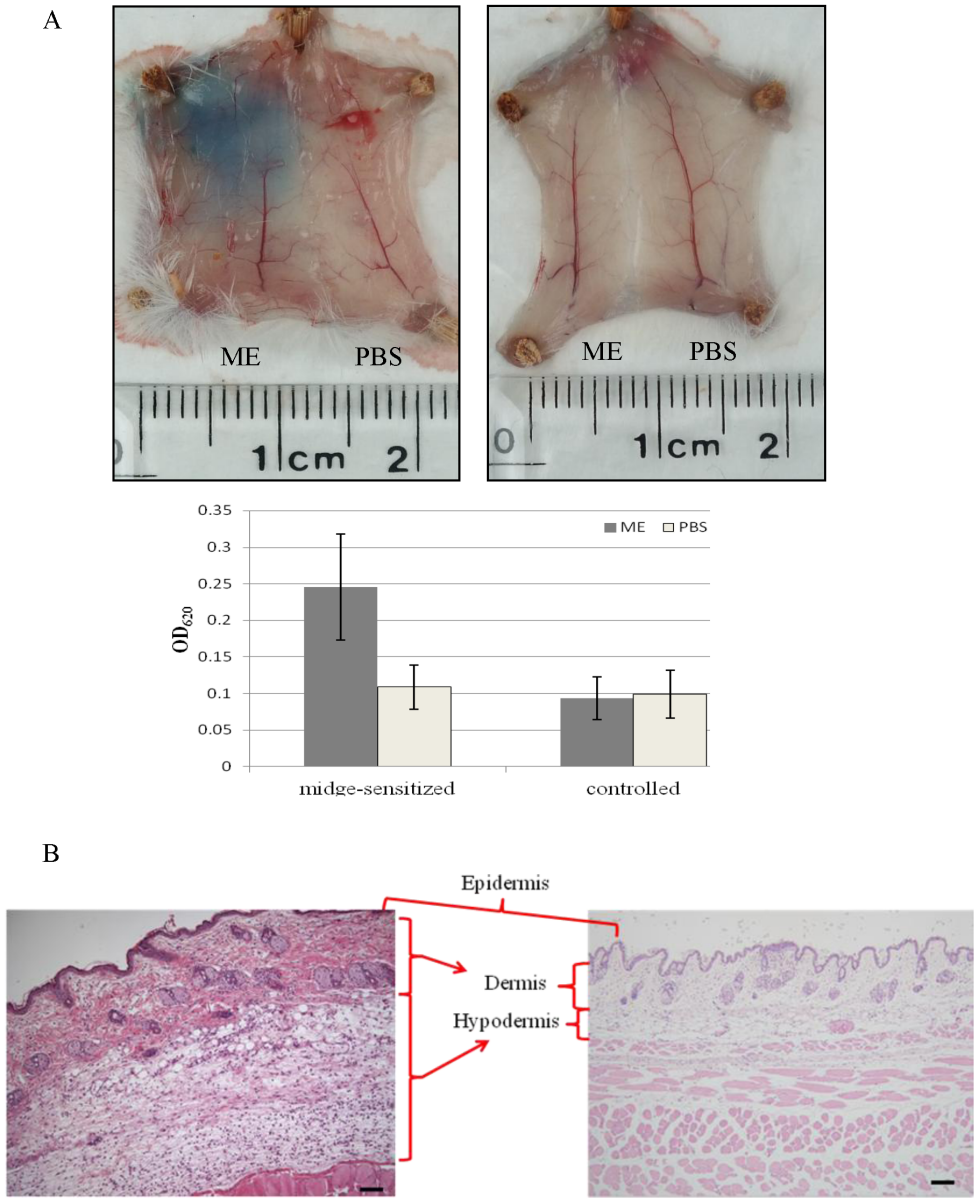
Representative skin biopsies of midge-sensitized or controlled mice. (A) Evans blue injection or (B) H and E-staining of abdominal skin sections from a midge-sensitized mouse (A, left and B) and a controlled mouse (A, right) after intra-dermal injection with PBS and midge extract (ME) at the indicated sides for 20 minutes. Magnification in B was ×100.

After the two-step sensitization, intra-dermal challenge with midge extract produced a marked increase in plasma extravasation in midge-sensitized mice, as revealed by Evans blue dye (Fig. 5A, left upper panel). In contrast, none of the control mice had plasma extravasation when challenged with midge extract (Fig. 5A, right upper panel). The amounts of extravasated dye were 4.76±1.75 vs. 1.08±0.73 μg/ml, respectively, between the two groups (n = 5, *p*<0.05) (Fig. 5A, lower panel).

Skin histology from midge-sensitized mice 20 min after midge extract challenge showed severe oedema and increased cellular infiltrations (Fig. 5B, left panel). However, no vascular change, dermal oedema, or cell infiltrates were observed in midge-sensitized PBS-challenged controls (Fig. 5B, right panel).

### Delayed Skin Reactions

**Figure 6:**
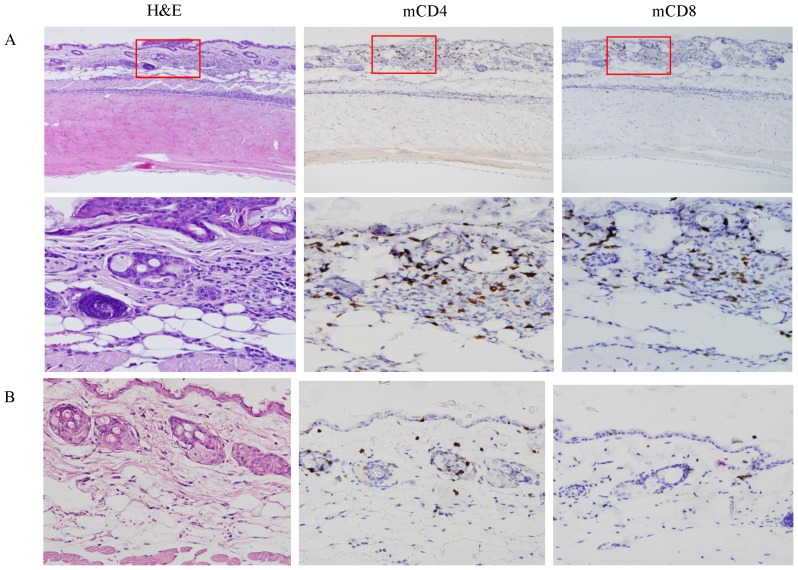
Histologic features of abdominal skins from (A) midge-sensitized and (B) controlled mice. The skin sections were obtained 48 hours after performing intra-dermal skin test and were stained with H and E or immuno-stained with anti-mouse CD4 (mCD4) or CD8 (mCD8) polyclonal antibodies. Magnification: A, upper, ×100; lower and B, ×400.

To further examine if this mouse model presented delayed-typed skin reactions to midge proteins, the skins from the challenged sites were biopsied 48 h after midge challenge. Skin biopsy from midge-sensitized mice showed marked leukocyte infiltration similar to that observed from human lesions (Fig. 6A, panel H and E). Immuno-histologic staining revealed higher numbers of murine CD4^+^ (mCD4) T cells of both the epidermis and dermis compared to the mCD8^+^ cells in midge-sensitized mice. These results are also similar to those of human skin lesions (Fig. 2). There were no increased inflammatory cells in the skin sections of the control mice (Fig. 6B).

### Midge-specific Splenocyte Proliferation and Cytokine Production

**Figure 7:**
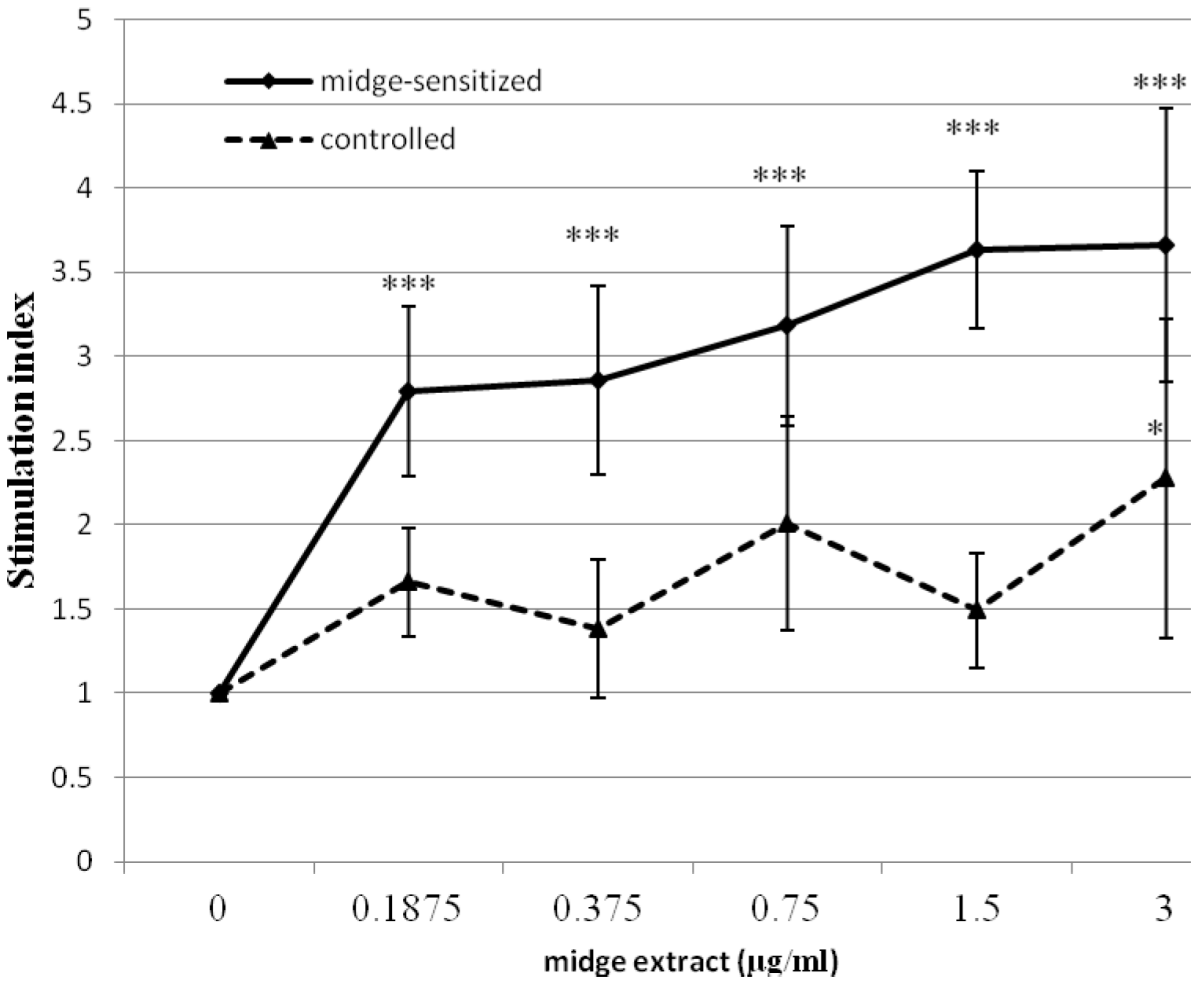
*In vitro* proliferation of midge-specific lymphocytes. Splenocytes from midge-sensitized or controlled mice were stimulated with increasing doses of midge extract for 3 days by MTT assay. The data are expressed as mean±SEM of the stimulation index (n = 5 per group). *p<0.05; ***p<0.001, by one-way ANOVA with the Dunnett *t* test.

To examine the effects of midge extract on mouse immune cells, splenocytes from sensitized and control mice were incubated with different concentrations of midge extract for 72 hours. The MTT assay revealed that the midge extract significantly induced a dose-dependent proliferation of splenocytes in midge-sensitized mice, but not in the control group (Fig. 7).

**Figure 8:**
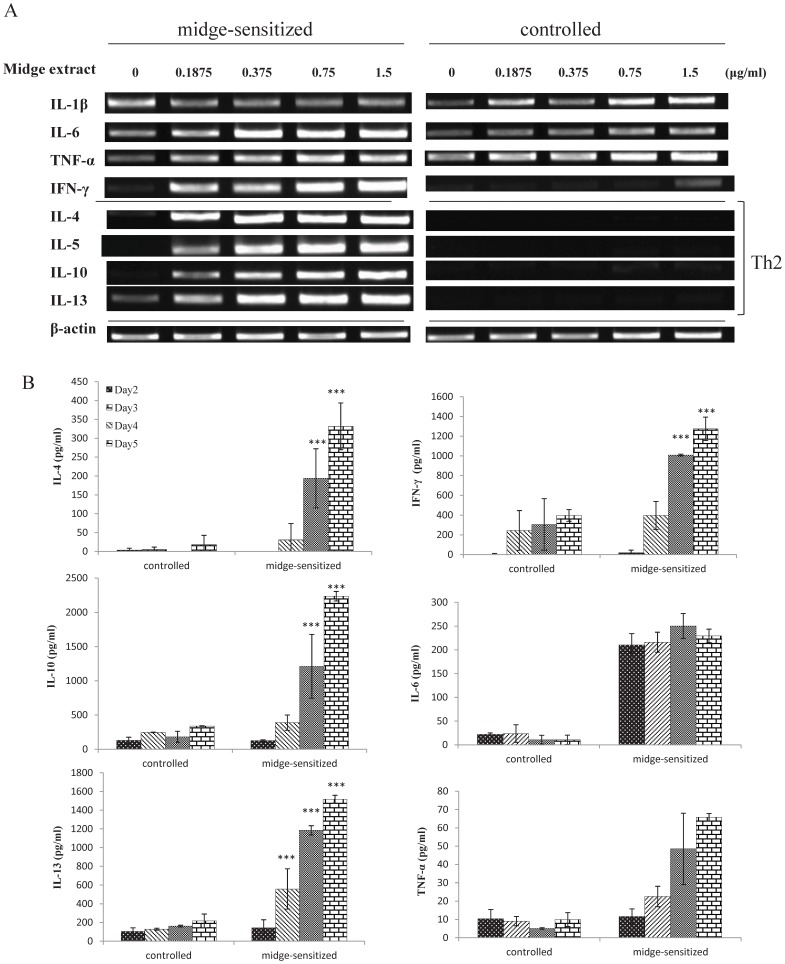
Cytokine expression levels of splenocytes from midge-sensitized or controlled mice. (A) Dose-dependent responses of midge extract on cytokine transcripts of splenocytes by RT-PCR. (B) Cytokine protein levels of splenocytes stimulated with 1.5 μg/ml of midge extract for 2–5 days. Cytokine data are expressed as means±SEM (n = 5 per group). *p<0.05; ***p<0.001, by one-way ANOVA with the Dunnett *t* test.

The kinetics of cytokine mRNA expression and protein levels in response to midge extract were determined by RT-PCR and ELISA, respectively. There was a marked up-regulation of mRNA expressions of IL-4, IL-5, IL-10, IL-13, and IFN-γ in midge-sensitized mice (Fig. 8A), but there were no differences in IL-1β, IL-6, and TNF-α mRNA expression between the two groups. The IL-4, IL-10, IL-13, IFN-γ, and TNF-α protein secretions were significantly elevated in the midge-sensitized mice in a time-dependent manner (Fig. 8B). IL-6 proteins were elevated since the first induction day in midge-sensitized mice, but not the control mice.

### Topical Application of the Dexamethasone Reduced the Delayed Inflammation

**Figure 9:**
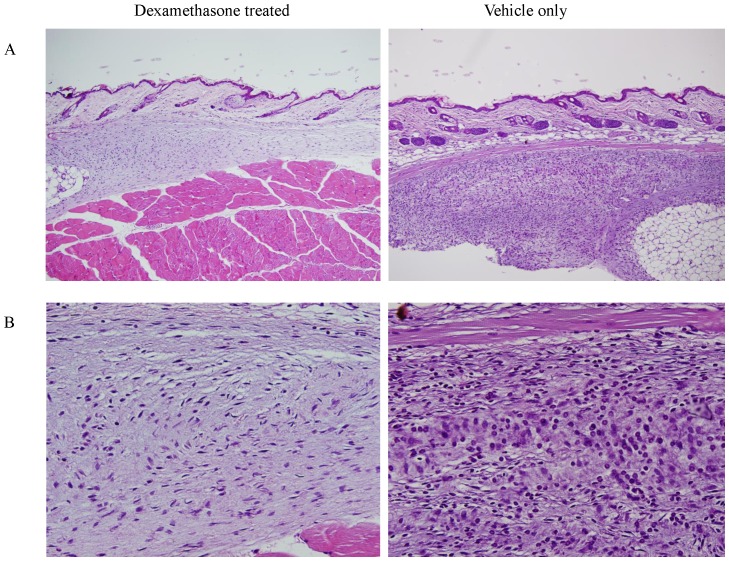
H and E staining show histologic features on midge-sensitized mice treated with dexamethasone ointment or with carbomer alone. Magnification: A, ×100; B, ×400.

Before the final ID challenge, topical dexamethasone or vehicle alone was topically applied daily for 1 week to the midge-sensitized mice. Histologic analysis revealed that skin from the dexamethasone-treated group had markedly reduced inflammatory cell infiltrations (Fig. 9A) compared to the skin of the carbomer vehicle-treated group (Fig. 9B).

## Discussion

Because of the stringent human-restricted blood sucking habit of the biting midge *Forcipomyia taiwana*, studies regarding this biting insect allergy remain scanty. Subjects with biting/sting insect allergy frequently avoid outdoor activities and this greatly impacts on their quality of life [7]. A validated animal model will be very helpful in studying this annoying problem. The present study developed a murine model of biting midge allergy using a two-step sensitization protocol involving an initial step of weekly intra-peritoneal injection of midge extract for four consecutive weeks, followed by a second step of intra-dermal injection of midge proteins every 3 or 4 days for 3 consecutive doses. The midge-sensitized mice demonstrated both immediate and delayed reactions to the midge challenge, as seen in human subjects. This model is unlike the mouse model for mosquito allergy [8], wherein the BALB/c mice were sensitized by exposing them to multiple mosquito (*Aedes aegypti*) bites, which is much closer to what happens naturally. However, as *Forcipomya taiwana* will not bite mice, the methodology cannot be the same here. There was also an attempt to skip the intra-peritoneal sensitization and simply sensitize the mice via intra-dermal route. However, the clinical and immune responses elicited by repeated intra-dermal sensitization were much less significant (data not shown) than that of the two-step protocol used here.

It is ideal to develop animal models for allergic diseases wherein the sensitization route is the same as the natural exposure route of human subjects [9]–[12]. However, this is not always possible. Sensitization through a non-natural route may lead to different disease manifestations, as what has been reported in the mouse model of visceral leishmaniasis between those transmitted by natural bites and intra-cardiac inoculation of parasites [13], the mouse model of peanut allergy that sensitized through oral and nasal routes [14], and the ovalbumin-induced anaphylaxis in mouse [15]. However, there are also reports whereby allergic reactions mimicking human allergic conditions can be induced via non-natural routes, as peanut allergy in sheep by injection with peanut extracts [16], cashew nut allergy by trans-dermal exposure [17], and many others [18]–[20]. More studies comparing different routes of allergen sensitization are warranted.

The mouse model for mosquito and sting insect allergy has been used to study the effects of anti-allergic treatment [21]–[24] and the mechanisms of allergic itch stimuli, as well as immune reactions to insect bites [25]–[30]. The mouse model described in this study may add more knowledge in these areas.

In conclusion, a murine model of midge bite allergy has been successfully developed using a two-step sensitization protocol. The sensitized mice have very similar clinical and immunologic reactions to challenge with midge proteins as the reactions of human to midge bites. This murine model may be a useful platform for future research and the development of treatment strategies for insect bite allergy.
